# Comprehensive integrated single-cell RNA sequencing analysis of brain metastasis and glioma microenvironment: Contrasting heterogeneity landscapes

**DOI:** 10.1371/journal.pone.0306220

**Published:** 2024-07-26

**Authors:** Seyedeh Fatemeh Sajjadi, Najmeh Salehi, Mehdi Sadeghi

**Affiliations:** 1 School of Biological Science, Institute for Research in Fundamental Sciences (IPM), Tehran, Iran; 2 National Institute of Genetic Engineering and Biotechnology (NIGEB), Tehran, Iran; University of Michigan Medical School, UNITED STATES

## Abstract

Understanding the specific type of brain malignancy, source of brain metastasis, and underlying transformation mechanisms can help provide better treatment and less harm to patients. The tumor microenvironment plays a fundamental role in cancer progression and affects both primary and metastatic cancers. The use of single-cell RNA sequencing to gain insights into the heterogeneity profiles in the microenvironment of brain malignancies is useful for guiding treatment decisions. To comprehensively investigate the heterogeneity in gliomas and brain metastasis originating from different sources (lung and breast), we integrated data from three groups of single-cell RNA-sequencing datasets obtained from GEO. We gathered and processed single-cell RNA sequencing data from 90,168 cells obtained from 17 patients. We then employed the R package Seurat for dataset integration. Next, we clustered the data within the UMAP space and acquired differentially expressed genes for cell categorization. Our results underscore the significance of macrophages as abundant and pivotal constituents of gliomas. In contrast, lung-to-brain metastases exhibit elevated numbers of AT2, cytotoxic CD4+ T, and exhausted CD8+ T cells. Conversely, breast-to-brain metastases are characterized by an abundance of epithelial and myCAF cells. Our study not only illuminates the variation in the TME between brain metastasis with different origins but also opens the door to utilizing established markers for these cell types to differentiate primary brain metastatic cancers.

## Introduction

The brain tissue environment exhibits significant distinctions from other tissues, making it uniquely poised to impact the low incidence of glioma brain metastasis, while also providing a more conducive environment for invasive cancers that reach the brain [1, 2]. Brain malignancies encompass two distinct categories of tumors: gliomas (GM) and brain metastasis (BM). GM refers to primary tumors that originate within the brain, whereas BM refers to secondary tumors that develop from primary tumors originating outside the brain, such as lung, breast, colorectal, and melanoma [3, 4]. Among central nervous system (CNS) cancers, BM stands as the most prevalent subtype [5–7], occurring at a high rate of 30% to 40% among individuals with cancer [8]. Differentiating between BM and GM poses considerable challenges due to their similar features and origins. Precisely distinguishing between the two forms of cancer is crucial, as their distinct treatment techniques greatly impact patient outcomes. Detecting metastasis early can facilitate identifying initial lesions in asymptomatic individuals [9–11]. The treatment approach for BM typically involves a combination of surgery, radiation therapy, and chemotherapy. The objective is to remove as much of the tumor as possible while preserving brain function and minimizing side effects. Additionally, targeted therapies might be employed in some cases for treating brain metastasis [12, 13]. While a combination of immune checkpoint blockade therapies has shown success in BM patients, not all individuals respond, presenting a therapeutic challenge [14]. BM represents a grave medical condition, and its prognosis varies based on factors such as the primary cancer type, metastasis size and location, and the patient’s overall health. The diagnosis, prognosis, and treatment selection for cancer are influenced by histological and phenotypic variances across different tumor types [15]. Nonetheless, individuals afflicted with metastatic brain tumors form a diverse group, making it challenging to predict the prognosis based solely on the origin of the primary tumor [16].

Tumors consist of diverse cell types, including immune and stromal cells, as well as regulatory molecules that impact tumor growth dynamics. Collectively, these constituents give rise to an intricate tumor microenvironment (TME) [12]. Moreover, the TME assumes a pivotal role in metastasis progression [17], and current research underscores its substantial influence on treatment response and clinical outcomes in cancer, warranting further investigation. Therefore, gaining a deeper insight into the TME landscape holds significant value. Recent studies have notably examined TME attributes in brain metastases, irrespective of their primary cancer source [3, 14].

Utilizing single-cell RNA sequencing (ScRNA-seq) represents a pragmatic approach in comprehending human cancers. This methodology enables the identification of cancer diagnostic biomarkers by analyzing the transcriptomes of individual cells and exploring the diversity within tumor cells [1, 12]. Progress in devising novel therapeutic strategies for brain metastases associated with diverse cancer types has been gradual, with recent breakthroughs focusing on targeting metastatic subclones and discerning selective niches. Earlier investigations primarily centered on evaluating the heterogeneity of glioma tumors and brain metastasis through single-cell RNA sequencing [1, 3, 4, 6, 8, 12, 17–21].

The brain’s TME is now widely recognized as a pivotal regulator of cancer, offering potential avenues for innovative treatment approaches [22]. Nevertheless, substantial research endeavors have recently been devoted to exploring the intricate interplay between TME heterogeneity and cancer progression. Many studies have delved into the diverse aspects of individual tumors’ heterogeneity [23–26].

Given the critical significance of early brain metastasis detection to enhance treatment outcomes, alongside the need to differentiate between gliomas and brain metastasis, our present study leveraged the tumor microenvironment’s heterogeneity and high-abundance cellular population markers to facilitate the identification and distinction of various brain tumor types. To illuminate the intricate nature of the TME, we integrated single-cell RNA sequencing data from human gliomas and brain metastasis originating from lung (BM-lung) and breast (BM-breast) tissues. Subsequently, we undertook a comparative analysis of the cellular and subtype heterogeneity across these integrated datasets. This analytical approach yielded a comprehensive insight into the profiles of brain metastases, gliomas, and the prognosis of primary cancers.

## Methods

### Sample collection and clinical characteristics

We sourced single-cell transcriptome data from various datasets: breast-to-brain metastasis (GSE186344, GSE143423), lung-to-brain metastasis (GSE186344, GSE131907, GSE143423), and glioma tumors (GSE117891, GSE202371, GSE135045) via the Gene Expression Omnibus (GEO) database. All samples included in our study comprised both patients with wild-type and mutated forms, as well as a mix of both males and females. Our glioma samples encompassed all three types: Astrocytoma, Oligodendroglia, and Ependymoma, across 4 grades as per the WHO classification [27]. The average age of glioma patients was 59 years. For patients with lung-to-brain and breast-to-brain metastases, the average ages were 57 and 53 years, respectively. Importantly, none of the surgical samples had undergone any prior treatment. Among patients with lung-to-brain metastasis, both histological types of Small Cell Lung Cancer (SCLC) and Non-Small Cell Lung Cancer (NSCLC) were represented. Similarly, among patients with breast-to-brain metastasis, both Triple-Negative Breast Cancer (TNBC) and Estrogen/Progesterone receptor positive (ER/PR+) types, including (human epidermal growth factor receptor 2) Her2-negative and Her2-positive subtypes, were included. All tumor tissue were obtained from the frontal lobe, cerebellum, parietal lobe, and temporal lobe. A total of 88,291 cells were acquired from 14 individuals utilizing the Chromium 10X Genomics scRNA-seq protocol across all three tumor groups. Additionally, 10,035 tumor cells from three glioma tumor samples that employed the Drop-Seq protocol, as detailed in the GSE135045 study, underwent analysis. All data collected in this study were mapped to the GRCh38 human reference genome.

### Processing of ScRNA-seq datasets

To uncover the common sources of biological variation we employed the integration tool for combining single-cell gene expression datasets using the R package Seurat version 4.1. We excluded cells of low quality, those with fewer than 200 expressed genes, and genes expressed in fewer than 3 cells from our analysis. Subsequently, cells with over 20% mitochondrial genes and those with excessively low or high gene counts were filtered out. Further, all datasets underwent standard preprocessing and were normalized through the LogNormalize method within the NormalizeData function of the Seurat package by a scale factor of 10000. Employing the variable feature variance stabilizing transformation method (selection.method = "vst"), we identified the top 2,000 highly variable genes across all samples using the FindVariableFeatures function of the Seurat package [28].

### Data integration and analysis

We utilized the anchor strategy to integrate datasets. Initially, we extracted anchors from the datasets and then proceeded to integrate them using 2000 anchors derived from the accurately identified anchor set. Next, scaling and Principal Component analysis (PCA) was executed on the integration dataset. To heuristically estimate the dataset’s dimensionality, we utilized Seurat’s Score JackStraw and elbow plot functions (S1A and S1B Fig). Subsequently, the first 35 principal components (PCs) were used to perform UMAP for nonlinear dimensional reduction. We applied a graph-based clustering approach from the Seurat package for clustering. Therefore we utilized the K-nearest neighbor (KNN) strategy with the FindNeighbors function and then used the FindClusters function to cluster with a resolution of 0.2. Also, other parameters set as Seurat default. Finally, we determined cell types by utilizing known markers for each cluster.

### Differential Expression Analysis of Genes (DEGs)

We conducted a differential expression analysis of genes using the non-parametric Wilcoxon rank-sum test provided by Seurat’s FindMarkers tool. This analysis aimed to identify genes that are expressed differently between the two groups of cells. For both cell groups, we set the minimum percentage (min.pct) threshold to 0.25. Additionally, we defined a threshold of 0.5 for the log fold-change in average expression (logfc.threshold) between the two cell groups.

To identify genes that were either upregulated or downregulated in each cluster, we calculated the average log fold-change values between that cluster and other clusters. This allowed us to quantify the extent of gene expression changes within each cluster

## Results

### Cell population analysis in glioma and lung-to-brain metastases

**Fig 1:**
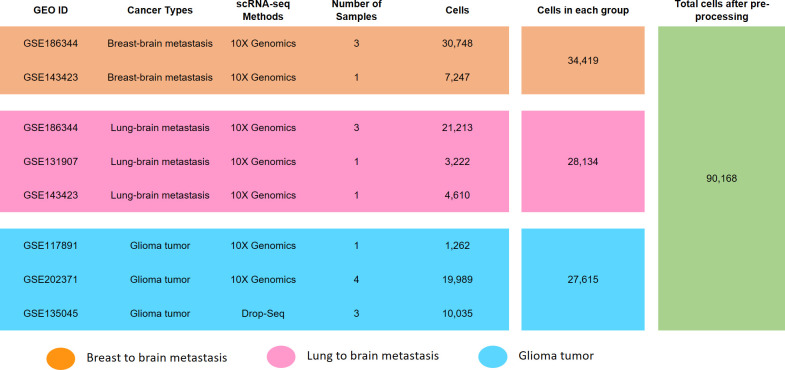
Datasets information of BM-lung, MB-breast, and GM.

In this study, we focused on BM originating from different types of cancers and compared these metastases with each other and GM. We utilized various human scRNA-seq datasets from GM and BM-lung, which were obtained from the GEO database. The details such as GEO ID, cancer type, scRNA-seq method, number of samples, and post-filtering cell count are presented in Fig 1.

**Fig 2:**
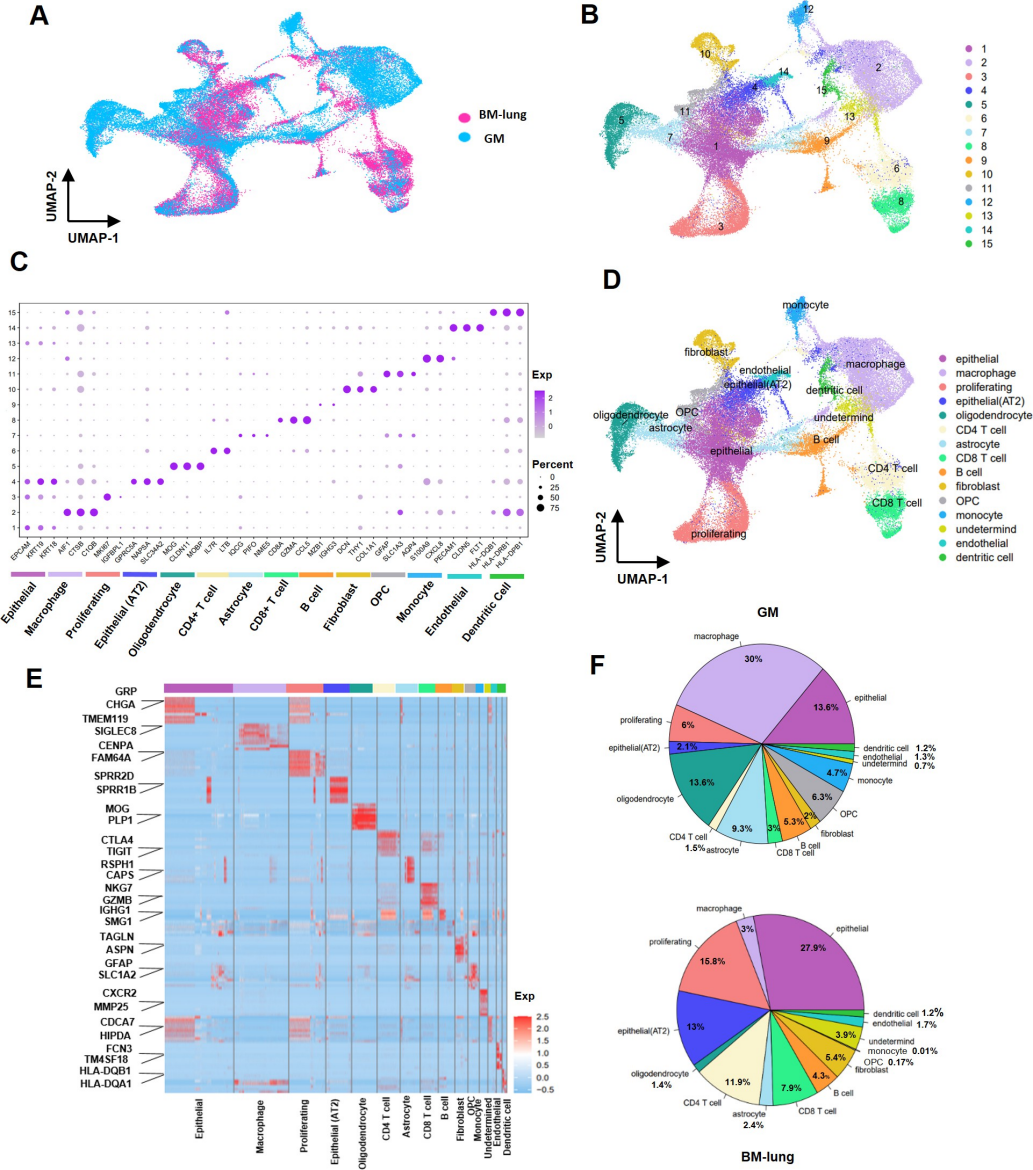
Transcriptional landscape of BM-lung and GM using 55,749 cells. (A) UMAP plot displaying 55,749 high-quality cells from 13 samples of BM-lung and GM, color-coded based on their original datasets. (B) UMAP plot presenting 55,749 high-quality cells from 13 samples of BM-lung and GM, color-coded by their clusters. (C) Assignment of cell types to clusters based on gene marker expression patterns in BM-lung and GM. (D) Dot plots illustrating conserved and cell-type-specific markers in BM-lung and GM. (E) Heatmap of gene expression levels of top-ranking marker genes in 15 different clusters. (F) Pie charts representing the percentage of each cell type in BM-lung and GM.

To perform a comparative analysis between GM and BM-lung, we pre-processed the transcriptomes of 60,331 distinct tumor cells originating from 8 GM and 5 BM-lung samples. We investigated the microenvironmental landscape and immunological state within GM and BM-lung. To achieve this, similar cell types across the various datasets were grouped together in a unified UMAP space, generated by integrating the datasets into a low-dimensional representation. The arrangement of each dataset within this integrated UMAP space is illustrated in Fig 2A. Using this integrated dataset, an unsupervised graph-based clustering approach identified a total of 15 distinct clusters of tumor cells. These clusters are visualized in the UMAP plot, as shown in Fig 2B. We identified a total of 15 distinct clusters, encompassing various cell types: epithelial cells (EPCAM, KRT19, KRT18), monocytes (S100A9, CXCL8), CD4+ T cells (IL7R, LTB), CD8+ T cells (CD8A, GZMA, CCL5), B cells (MZB1, IGHG3), astrocytes (IQCG, PIFO, NME5), macrophages (AIF1, CTSB, C1QB), alveolar type 2 epithelial cells (AT2) (GPRC5A, NAPSA, SLC34A2), proliferating cells (MKI67, IGFBPL1), oligodendrocytes (MOG, CLDN11, MOBP), fibroblasts (DCN, THY1, COL1A1), oligodendrocyte precursor cells (OPC) (GFAP, SLC1A3, AQP4), dendritic cells (HLA-DQB1, HLA-DRB1, HLA-DPB1), endothelial cells (PECAM1, CLDN5, FLT1), and undetermined cells (depicted in Fig 2C) [6, 21, 29–36]. The top 100 up-regulated genes for each cluster are listed in S1 Data, based on the Differential Expression Analysis. Our findings highlight significant differences in cell population composition between BM-lung and GM. Notably, there were noteworthy distinctions in the abundance of immune cell types. Macrophages (30% in GM, 3% in BM-lung) and monocytes (4.7% in GM, 0.01% in BM-lung) were notably more abundant in GM, whereas BM-lung exhibited a higher frequency of CD4+ T (11.9% in BM-lung, 1.5% in GM) cells and CD8+ T cells (7.9% in BM-lung, 3% in GM). Moreover, the prevalence of CNS cells, including oligodendrocytes (13.6% in GM, 1.4% in BM-lung), astrocytes (9.3% in GM, 2.4% in BM-lung), and OPC (6.3% in GM, 0.17% in BM-lung), was more pronounced in GM. In contrast, epithelial cells, particularly of the AT2, were more prevalent in BM-lung (13% in BM-lung, 2.1% in GM) (S1 Table in S1 File). A distinct cluster contained cell types that could not be definitively determined. Canonical markers for each cell type in BM-lung and GM are depicted in Fig 2C, and determined clusters show in UMAP space in Fig 2D. The 10 top genes expressed in each clusters show in heatmaps (Fig 2E). The distribution of cell percentages in BM-lung and GM is visualized in Fig 2F.

### Comparative analysis of immune cell sub-clusters in glioma and lung-to-brain metastasis

**Fig 3:**
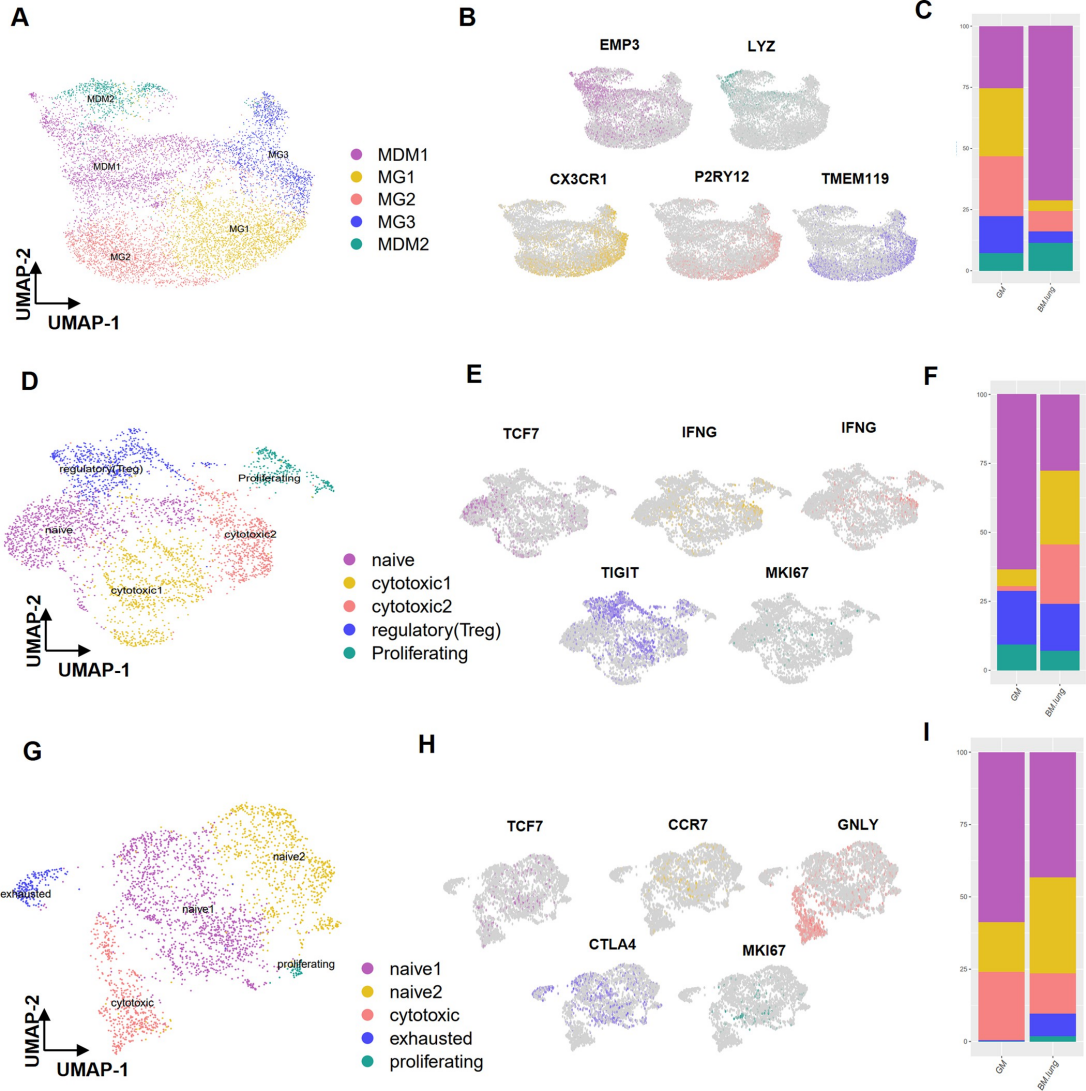
Sub-clustering of immune cells in the transcriptional landscape of BM-lung and GM. (A) Sub-clustering of macrophages and the identification of two clusters of microglia and MDM (UMAP plot). (B) Feature plot displaying the marker expression of three marker genes for each sub-cluster of macrophages. (C) Distribution of macrophage sub-clusters between GM and BM-lung in bar plot. (D) Sub-clustering of CD4+ T cells depicted in the UMAP plot. (E) Feature plot illustrating the marker expression of three marker genes for each sub-cluster of CD4+ T cells. (F) Distribution of CD4+ T cell sub-clusters between GM and BM-lung in bar plot. (G) Sub-clustering of CD8+ T cells shown in the UMAP plot. (H) Feature plot displaying the marker expression of three marker genes for each sub-cluster of CD8+ T cells. (I) Distribution of CD8+ T cells sub-clusters between GM and BM-lung in bar plot.

To gain a deeper understanding of the immune cell subpopulations, we conducted targeted analyses. Specifically, we focused on macrophages and T cells within these subpopulations. By employing the FindClusters function, we isolated five distinct subclusters within macrophage cells. Through gene expression markers, we successfully delineated two primary cell types (illustrated in Fig 3A). In the brain’s tumor microenvironment, tumor-associated macrophages (TAM) were found to exist in two sub-clusters: monocyte-driven macrophages (MDM) (identified by EMP3, ACP5, and LYZ markers) and microglia (MG) (characterized by CX3CR1, TMEM119, and P2RY12, markers) (depicted in Fig 3B) (S2A and S2B Fig) Among these sub-clusters, sub-clusters 2, 3, and 4 were dominated by MG and exhibited higher prevalence in GM. Conversely (67.3% in GM and 17.4% in BM-lung), sub-clusters 1 and 5 were identified as MDM and were more abundant in BM-lung (82.5% in BM-lung and 32.6% in GM) (S2 Table in S1 File). Also, percentages of each subclusters show in bar plot (Fig 3C). Furthermore, our investigation extended to CD4+ T cells, which were categorized into five sub-clusters using the FindClusters function. These sub-clusters encompassed naive CD4+ T cells (marked by TCF7, CCR7), cytotoxic CD4+ T cells (characterized by IFNG, GZMM, GZMA, GNLY), regulatory (Treg) CD4+ T cells (identified by TIGIT, CTLA4, IL2RA), and proliferating cells (indicated by MKI67, RRM2, TYMS) (displayed in Fig 3D). Notably, sub-clusters 2 and 3 exhibited a higher prevalence in BM-lung, and further analysis confirmed that both of these sub-clusters comprised cytotoxic CD4+ T cells) (48.2% in BM-lung and 7.7% in GM) (shown in S2C and S2D Fig) (S3 Table in S1 File). UMAP plots with well-established cell type-specific marker genes are depicted in Fig 3E. Distribution of cell type percentages in CD4+ T cell is visualized in Fig 3F. Within CD8+ T cells, we identified five sub-clusters (Fig 3G) (S2E and S2F Fig). Sub-cluster 4 exhibited greater prevalence in BM-lung compared to GM, representing exhausted CD8+ T cells (noted by CTLA4) (7.6% in BM-lung and 0.4% in GM). Another sub-cluster within CD8+ T cells demonstrated relatively consistent population proportions between BM-lung and GM, encompassing naive CD8+ T cells (marked by IL7R, TCF7, CCR7), cytotoxic CD8+ T cells (characterized by GNLY, FGFBP2), and proliferating CD8+ T cells (indicated by MKI67, TPX2) (G9) (Fig 3H) (S4 Table in S1 File). Percentages of cell types in CD8+ T cell is visualized in Fig 3I.

### Cellular population analysis in breast-to-brain metastases and glioma

**Fig 4:**
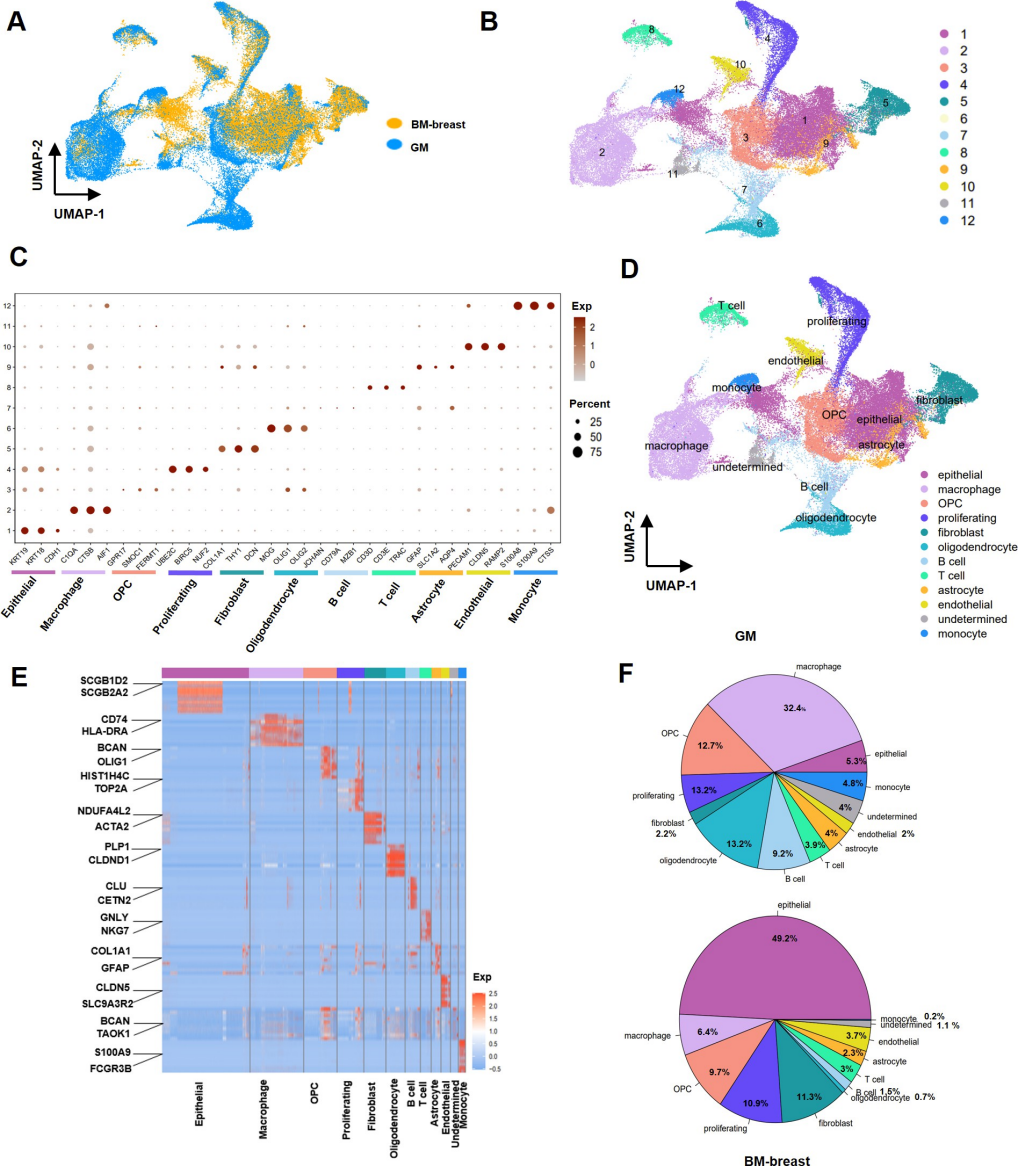
Transcriptional landscape of BM-breast and GM using 69,785 cells. (A) UMAP plot displaying 69,785 cells from 12 samples of BM-breast and GM, color-coded according to their original datasets. (B) UMAP plot presenting 55,749 high-quality cells from 12 samples of BM-breast and GM, color-coded by their clusters. (C) Assignment of cell types to clusters based on gene marker expression patterns in BM-breast and GM. (D) Dot plots illustrating conserved and cell-type-specific markers in BM-breast and GM. (E) Heatmap of gene expression levels of top-ranking marker genes in 12 different clusters. (F) Pie charts representing the percentage of each cell type in BM-breast and GM.

In order to elucidate the cellular composition within the tumor microenvironment of BM-breast and GM, we conducted a comprehensive investigation involving three BM-breast samples and eight GM samples. To assess heterogeneity, we meticulously processed and analyzed data from a total of 69,281 tumor cells across the datasets. The arrangement of each dataset within the integrated UMAP space is depicted in Fig 4A. Employing clustering techniques, we categorized the cells into 12 distinct clusters (Fig 4A and 4B). We assigned these clusters based on the expression of established marker genes, as illustrated in Fig 4C. The identified cell types included T cells (marked by CD3E, CD3D, TRAC), B cells (characterized by JCHAIN, MZB1, CD79A), OPC (identified by GPR17, SMOC1, FERMT1), macrophage cells (noted by C1QA, CTSB, AIF1), monocyte cells (characterized by CTSS, S100A9, S100A8), proliferating cells (indicated by UBE2C, BIRC5, NUF2), endothelial cells (marked by RAMP2, CLDN5, PECAM1), fibroblasts (distinguished by DCN, COL1A1, THY1), oligodendrocytes (characterized by MOG, OLIG1, OLIG2), astrocytes (noted by GFAP, SLC1A2, AQP4), and undetermined cells (Fig 4D) [25, 37–43]. The top 100 up-regulated genes for each cluster are provided in S2 Data.Our findings indicated a greater prevalence of epithelial (49.2% in BM-breast and 5.3% in GM) and fibroblast (11.3% in BM-breast and 2.2% in GM) cells in BM-breast compared to GM. Conversely, myeloid cells (macrophages and monocytes) (37.1% in GM and 6.5% in BM-breast), B cells (9.2% in GM and 1.5% in BM-breast), oligodendrocytes (13.2% in GM and 0.7% in BM-breast), and undetermined cells were more abundant in GM. The 10 top genes expressed in each clusters show in heatmaps (Fig 4E). The distribution of cell percentages in BM-breast and GM is displayed in Fig 4F (S5 Table in S1 File).

### Sub-clustering of macrophage and fibroblast cell types

**Fig 5:**
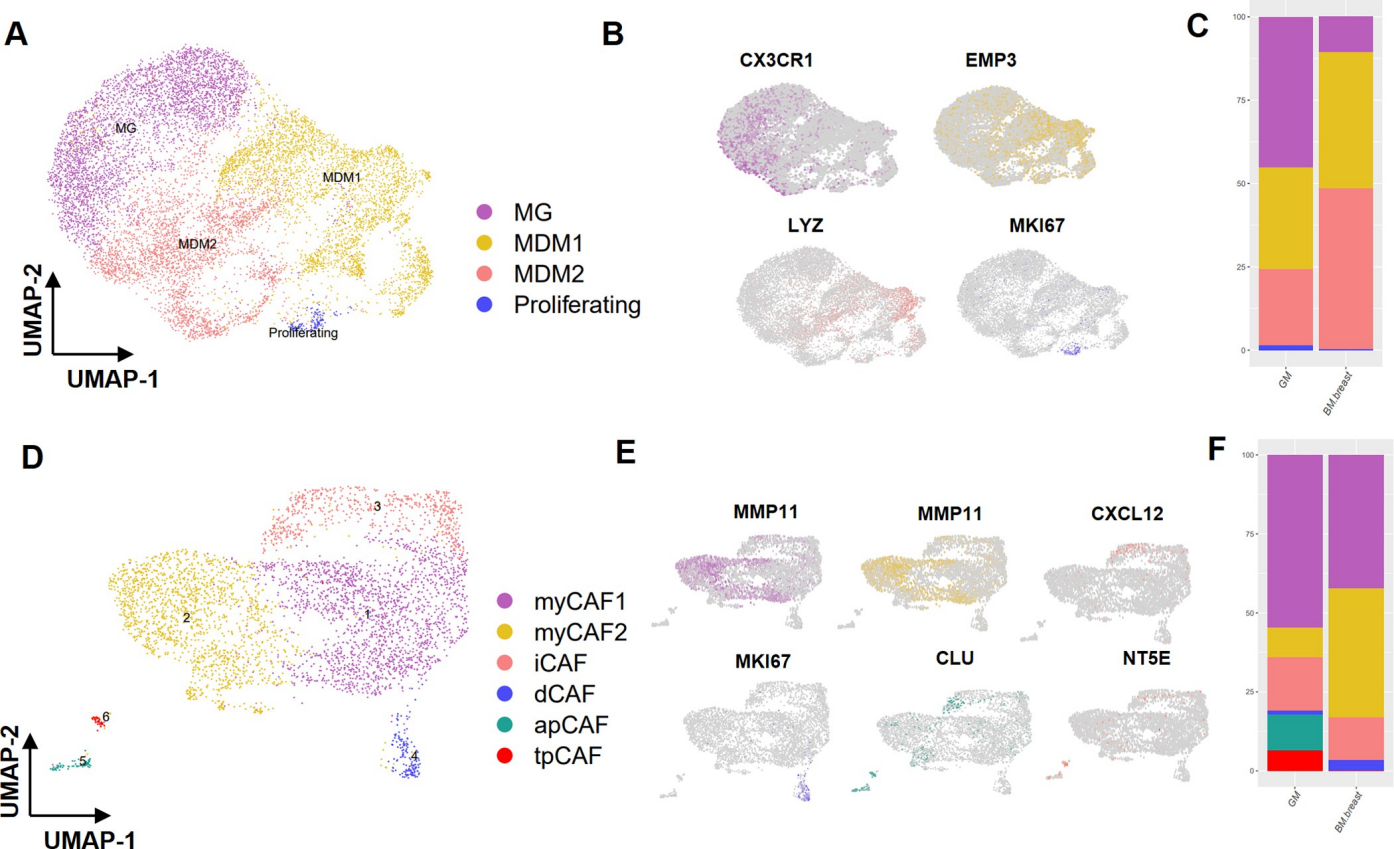
Sub-clustering of macrophage and fibroblast cells in the transcriptional landscape of BM-breast and GM. (A) UMAP plot displaying the sub-clustering of macrophages and the identification of two clusters of MG and MDM. (B) Feature plot illustrating the marker expression of three marker genes for each sub-cluster of macrophages.(C) Distribution of CD4+ T cell sub-clusters between GM and BM-breast in bar plot. (D) UMAP plot showcasing the sub-clustering of CAFs. (E) Feature plot displaying canonical marker genes for each sub-cluster of CAFs. (F) Distribution of CAFs cell sub-clusters between GM and BM-breast in bar plot.

To gain deeper insights into the subtypes within macrophages and fibroblasts, we undertook sub-clustering of these cell type groups (S3A and S3B Fig). Within the macrophage cell population, sub-clustering revealed three distinct subgroups: MG, MDM, and proliferating cells (Fig 5A). MG cells were notably more abundant in GM compared to BM-breast (45.2% in GM and 10.5% in BM-breast). Notably, the expression of well-known gene markers CX3CR1 and EMP3, MKI67 allowed accurate classification of MG, MDM, and proliferating subtypes, respectively (Fig 5B) (S6 Table in S1 File).

Fibroblasts in the tumor context, specifically Cancer-Associated Fibroblasts (CAFs), constitute a heterogeneous cell population with diverse roles in the tumor microenvironment. In order to comprehensively comprehend the heterogeneity of CAFs, we conducted sub-clustering of fibroblast cell types. This analysis revealed six distinct phenotypes within the fibroblast cluster (Fig 5D) (S3C and S3D Fig). By analyzing gene expression markers, we successfully identified five cell types, namely inflammatory-like CAFs (iCAFs) (characterized by CXCL12, IL6), myofibroblast-like CAFs (myCAFs) (marked by ACTA2, COL1A1, MMP11), dividing CAFs (dCAFs) (identified by TUBA1B, MKI67), antigen-presenting CAFs (apCAFs) (noted by CD74, TMEM158, CLU), and tumor-promoting CAFs (tpCAFs) (characterized by TN5E, PDPN5, VEGFA) (Fig 5E) [43–46]. It is noteworthy that apCAFs (11.1% in GM and 0% in BM-breast) and tpCAFs (6.5% in GM and 0% in BM-breast) displayed higher abundance in GM, while myCAFs were more prevalent in BM-breast (40.7% in BM-breast and 9.5% in GM) (S7 Table in S1 File). Percentages of cell types in CD8+ T cell is visualized in Fig 3F.

### Validation of results

To assess the accuracy and practicality of our derived findings, we conducted a validation using two samples from the GEO database one GM sample (GSM3984326 from GSE135045) and one BM-lung sample (GSM6112137 from the GSE202371 dataset). These samples yielded a total of 88,291 cells from two individuals, which were subsequently processed and filtered down to 8,413 cells. Employing the same integration methodology outlined in the method section, the datasets were integrated.

**Fig 6:**
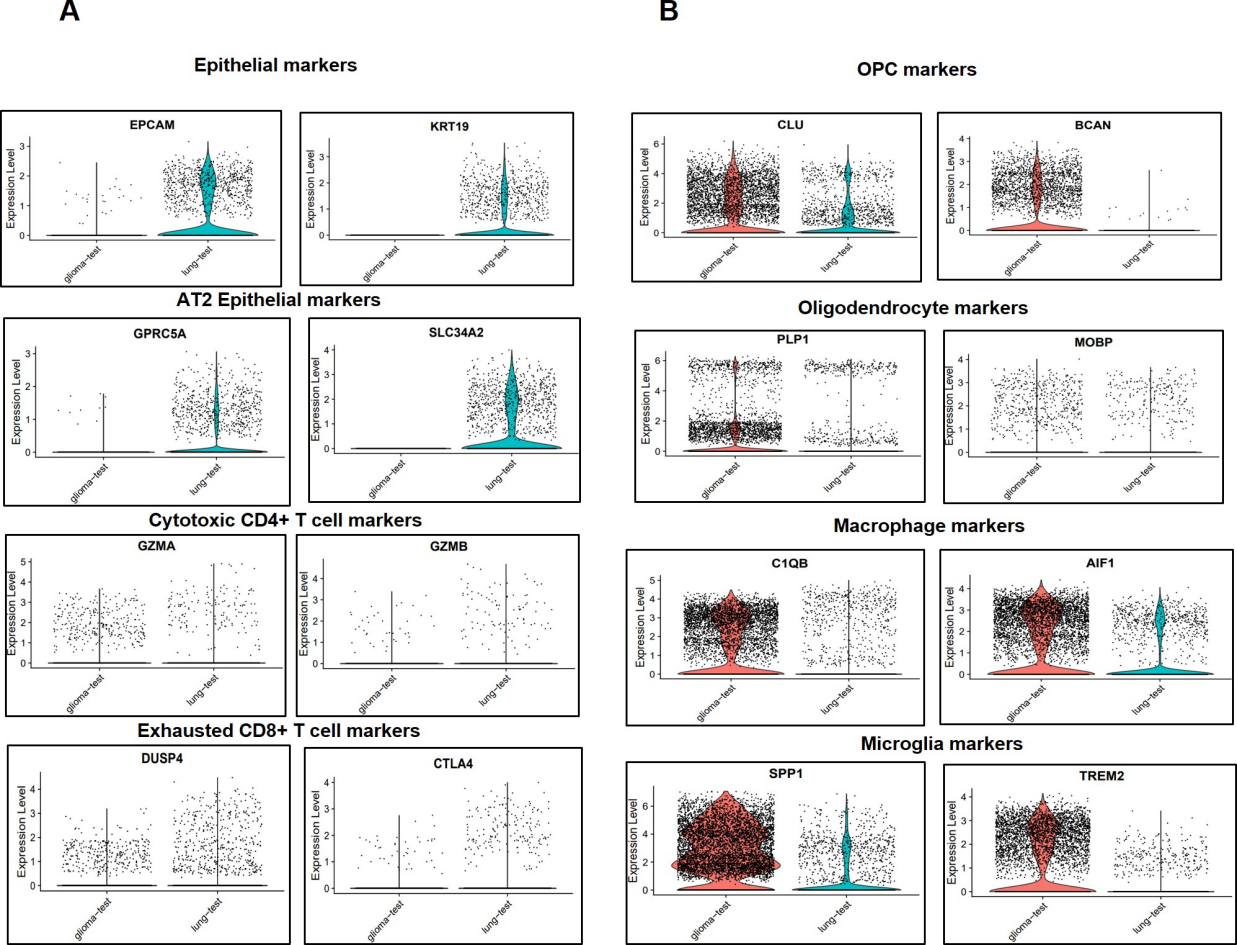
Expression levels of canonical marker genes for cell types in BM-lung and GM test samples. (A) Expression levels of canonical marker genes with higher expression in BM-lung. (B) Expression levels of canonical marker genes with higher expression in GM.

Post-integration, we scrutinized the expression of marker genes for various cell types. Remarkably, our validation results echoed the findings obtained from the comparison between BM-lung and glioma. The expression markers for cell types including T cells, exhausted CD8+ T cells, cytotoxic CD4+ T cells, macrophages, MG, and AT2 epithelial cells were evaluated. Notably, cytotoxic CD4+ T cells, exhausted CD8+ T cells, and AT2 epithelial cells exhibited higher abundance in BM-lung (Fig 6A). In contrast, macrophages, microglia, OPCs, and oligodendrocytes were more prevalent in GM (Fig 6B).

## Discussion

BM represents a pressing healthcare concern in oncology treatment. Given the bleak prognoses associated with primary cancers that have spread to the brain, especially lung and breast cancers, there is an urgent imperative to enhance our understanding of the underlying pathogenic mechanisms and uncover novel targets for immune-based therapies [16]. It has become increasingly evident that the TME within the brain plays a pivotal role in shaping cancer progression and the efficacy of treatments, both for primary brain tumors and metastatic lesions. Mechanistic insights into the tumor-promoting activities of various constituents within the brain TME have unveiled multiple potential targets for therapeutic intervention [23, 47].

In recent years, significant research endeavors have been dedicated to exploring the intricate interplay between the immune system and the TME in BM. This exploration has led to a paradigm shift, recognizing the CNS as an immunologically distinct domain, as opposed to an isolated one [22]. Furthermore, advancements in scRNA-seq have facilitated a comprehensive analysis of tumor and immune microenvironment heterogeneity across various cancer types [15]. To unravel the intricacies of the tumor microenvironment in both BM and GM, we meticulously analyzed extensive scRNA-seq datasets encompassing lung and breast-to-brain metastasis and compared the diversity of cellular constituents between these entities.

Our focus centered on profiling the distinct cell lineages that coalesce within tumors, encompassing immune cells, oligodendrocytes, endothelial cells, epithelial cells, and fibroblasts. Following cluster assignment, we unveiled a unique subset of AT2 epithelial cells within BM-lung, exhibiting a notably lower prevalence in glioma and BM-breast. Previous research has underscored the significance of AT2 cells as crucial players in lung cancer origin and their potential role in facilitating lung cancer metastasis [24, 48]. Hence, this specific cluster and its distinct gene expression markers (GPRC5A, NAPSA, and SLC34A2) hold promise as prognostic indicators for lung cancer, a prominent source of brain metastases [33, 34].

Tumor cells can incite an immune response, leading to a complex equilibrium where diverse immune subtypes can either foster tumor progression, and metastasis, or confer resistance to treatments [49, 50]. Among the key elements in the microenvironment contributing to immune evasion are the expansion of pro-inflammatory macrophages and the malfunctioning of T cells. Macrophages, known for their role in maintaining tissue homeostasis, have garnered increasing attention in the tumor microenvironment of brain tumors. Interestingly, when influenced by cancer cells, macrophages tend to polarize into immune suppressor cells, thereby promoting an environment conducive to tumor growth and evasion of immune surveillance [22, 51, 52].

Our findings highlight the prevalence of myeloid cells, specifically monocytes and macrophages, as the predominant immune cell types in GM. Prior research has illuminated the dynamic roles played by myeloid cells in cancer, wherein their functions range from exhibiting anti-tumor activities to promoting tumor growth, contingent upon the cancer type and its stage [42, 53]. Furthermore, existing studies have pointed out distinctions between the uptakes rates of macrophages in BM compared to GM. Despite functional similarities between these two diseases, differences in their microenvironments persist [26, 54]. Given the pivotal role of TAMs in orchestrating tumor progression, targeted interventions are gaining traction as a strategy to disrupt the tumor-promoting activities of TAMs [54]. In our sub-clustering analysis of TAMs within GM, BM-lung, and BM-breast, noteworthy patterns emerged. Specifically, the MG subtype exhibited higher prevalence in GM compared to BM-lung and BM-breast. In contrast, the MDM subtype was more abundant in BM-lung and BM-breast. Prior investigations have established that MDM is implicated in processes such as antigen presentation, immune suppression, and wound healing, whereas MG is associated with tasks related to host defense mechanisms and maintenance activities like synaptic pruning [26, 37, 55].

Considering the distinct roles played by each of the two subtypes in BM and GM, comprehending the presence of their respective populations within these conditions can significantly assist in tailoring treatments based on the immune cell landscape. In the transcriptional profiles of BM-breast and GM, we have identified elevated frequencies of epithelial and fibroblast cell types. Conversely, in GM cases, heightened frequencies were observed among myeloid cells, B cells, and oligodendrocytes. Earlier studies on BM have indicated that patients with BM-breast exhibit increased frequencies of macrophages, whereas cases of lung cancer demonstrated higher T cell frequencies [8].

Consistent with these prior findings and in alignment with our discoveries, the immune cell clusters and frequencies within BM differ based on the cancer’s point of origin. These disparities have the potential to influence targeted therapeutic approaches for BM [8, 21]. Upon closer examination of the immune cell subgroups, we have noted that elevated macrophage levels in GM, rather than BM, are linked to MG. MG, often referred to as brain macrophages, play a crucial role in immune regulation and the elimination of tumors [8]. Sub-analysis of the T cell landscape reveals that T cells (both CD4+ and CD8+) dominate the immune cell composition in BM-lung compared to GM. When investigating the sub-clusters within T cell lymphocytes in BM-lung and GM, it becomes apparent that cytotoxic CD4 T cells (characterized by GZMM, GZMA, GZMB, IFNG, and GNLY) and exhausted CD8+ T cells (marked by CD8A, CTLA4, LAG3, and TIGIT) [33] exhibit a high frequency in BM-lung. Previous studies on brain metastasis have shown a higher population of leukocytes in brain metastasis than in CNS-endogenous cancers [55–58].

Within the realm of immunotherapies, the establishment of a lasting response is limited to a subset of patients, primarily due to the tumor microenvironment’s suppressive effects on the immune system. It’s worth noting that CD4+ T cells identify distinct surface markers compared to CD8+ T cells, and given that cancer cells generally lack MHC-II expression, CD4+ T cells demonstrate effectiveness in exerting tumor suppression through interactions with stromal cell surface markers. For instance, these interactions involve macrophages, B cells, and the release of cytokines to facilitate CD8+ T cell activation. Post antigen encounter, a significant portion of T effector cells undergo apoptosis, leading to the expansion of the exhausted T cell phenotype within cytotoxic effector populations in CD8+ T cell subsets [31, 52, 59, 60].

CD8+ T cells function as cytotoxic lymphocytes responsible for detecting and responding to infections. Following pathogen elimination, effector CD8+ T cells transition into memory cells that provide protection upon subsequent exposure to antigens. However, in the context of malignancy, effector CD8+ T lymphocytes experience exhaustion, resulting in diminished proliferative and cytotoxic capabilities [61–63]. Recent investigations underscore that T cell exhaustion and functional impairment within the TME are fundamental features of various malignancies. These alterations in immune cell populations contribute to the transformation of the tumor milieu into an immunosuppressive environment effectively establishing immunosuppressive conditions within the tumor tissue [31, 59]. Collectively, this data highlights the substantial role played by the tumor’s origin in shaping the specific characteristics of the brain metastatic tumor microenvironment.

Cancer-associated fibroblasts (CAFs) constitute the predominant component of the tumor microenvironment. As the tumor grows, the healthy breast matrix undergoes disruption, marked by a reduction in the number of healthy fibroblasts and their conversion into CAFs. CAFs release various cytokines, chemokines, extracellular matrix regulatory molecules, components of the extracellular matrix, and inflammatory mediators, collectively promoting tumor cell proliferation, invasion, metastasis, and evasion from immune surveillance [37, 39]. These CAFs contribute to the TME by generating enzymes that crosslink the matrix and proteases that degrade the extracellular matrix, significantly contributing to increased tissue stiffness and facilitating metastasis [64, 65].

Numerous studies have confirmed the presence of diverse phenotypic and functional CAF populations within a single tumor, observed both in vivo and in vitro, along with single-cell analyses of pre-sorted CAFs [64, 66–70]. Importantly, this study represents the first attempt to compare CAF subpopulations between BM-breast and GM. Through our analysis of single-cell RNA sequencing data, we have identified six distinct CAF subtypes based on their transcriptome profiles. The allocation of cells within these sub-clusters reveals higher levels of apCAFs and tpCAFs in GM, while myCAFs predominate in BM-breast. Previous research has unveiled that myCAFs actively produce a range of matrix components and participate in matrix remodeling. They also secrete cytokines and chemokines, along with inflammatory factors that facilitate tumor cell adhesion and migration. On the other hand, tpCAFs express metalloproteinases and matrix proteins, which contribute to extracellular matrix remodeling [45]. Additionally, dCAFs exhibit a more specialized expression pattern, specializing in the production of basement membrane components and paracrine signaling molecules [41, 71]. Earlier investigations have indicated that GM do not exhibit a high abundance of CAFs; however, they do accumulate within the GM microenvironment. These CAFs develop a more mesenchymal phenotype, contributing to enhanced migratory and invasive behaviors in malignant cells [72–74].

Chemotherapy is a frequent treatment for cancers and can significantly impact the disease process. Cancer cells’ treatment sensitivity is heavily influenced by their interactions with the tumor microenvironment (TME), especially immune cells. There’s currently significant interest in targeting stromal cells in cancer therapy [75–77]. It’s crucial to accurately assess the composition of stromal cells in tumors, replicate the diverse characteristics seen in human tumors in clinical models, and understand how this diversity impacts treatment efficacy, drug responses, and resistance [78, 79]. Clinical studies indicate a correlation between CD4, CD8, and macrophage levels in the tumor microenvironment and treatment outcomes. The immune system plays an active role in illness, potentially affecting clinical responses and resistance to treatment. An abundance of macrophages can hinder therapeutic effectiveness. For instance, in patients with node-positive breast cancer who underwent intensive chemotherapy, those with tumors exhibiting high levels of macrophages, high CD4 T-cells, but low CD8 T-cells, experienced significantly lower recurrence-free survival rates compared to those with tumors showing low macrophage levels, low CD4 T-cells, and high CD8 T-cells. In addition, Cytotoxic T-cells play a positive role in treatment effectiveness [80–82].

Furthermore, cancer-associated fibroblasts (CAFs) play a crucial role in the treatment process due to their increased proliferation, enhanced extracellular matrix production, and unique cytokine secretion compared to normal tissue fibroblasts. Early co-culture studies suggested that injured or irradiated fibroblasts might promote cancer cell proliferation more effectively than non-irradiated fibroblasts, indicating that within a solid tumor, fibroblasts are not passive entities and could potentially influence therapy outcomes [81, 83, 84].

Researchers identified components produced by normal human fibroblasts in a preclinical model of genotoxic damage, and discovered that WNT-16b might drastically restrict tumor response through paracrine signaling. In a WNT-dependent way, elevated amounts of this ligand promoted the growth of cancer cells and produced a mesenchymal phenotype. The responsiveness of fibroblasts to chemotherapy was enhanced by removing WNT-16b. Stromal fibroblasts secreted WNT-16b through an NF-κB-mediated pathway linked to inflammation and stress. Because stress-response programs in stromal cells might decrease treatment efficacy by providing a protective environment for cancer cells, the supporting stroma’s reaction to therapy may be more complicated [84].

The study has several limitations, including potential variability from integrating single-cell RNA sequencing datasets from different sources such as lung-to-brain metastasis, breast-to-brain metastasis, and gliomas, which may introduce batch effects despite using the anchor technique for data harmonization. Additionally, the sample size of both patients and cells analyzed may limit the statistical power and comprehensive representation of heterogeneity within gliomas and brain metastases. Furthermore, the findings, based on a specific patient cohort, may not fully represent broader populations or diverse clinical settings, thus limiting the generalizability of the results. The identified cell types and their associations with disease progression or therapeutic response are observational and require further validation through functional studies and clinical trials.

## Conclusion

In conclusion, the prevalence of distinct cell types exhibiting varying population proportions in BM arising from different primary cancers holds the potential for aiding in the prediction of the primary brain cancer type. Our findings underscore the significance of macrophages as abundant and crucial elements within GM. Conversely, MB-lung exhibit elevated populations of AT2 cells, cytotoxic CD4+ T cells, and exhausted CD8+ T cells. Meanwhile, BM-breast are characterized by an abundance of epithelial cells and myCAFs. Our study not only sheds light on the heterogeneity of the TME between BM-lung and BM-breast cases but also introduces the possibility of leveraging well-known markers for these cell types to distinguish primary brain metastatic cancers. Looking ahead, it remains imperative to discern the nuanced variations in microenvironmental composition across diverse brain tumor subtypes to attain a comprehensive comprehension of tumor biology. Consequently, enhancing our understanding of the tumor microenvironment contributes to the identification of primary tumors within brain metastasis scenarios, thereby facilitating the selection of more tailored treatment approaches based on the originating primary cancers.

## Supporting information

S1 Fig Elbow plot. Elbow plot of BM-lung and GM (A) Elbow plot of BM- breast and GM (B).
(TIF)

S2 Fig UMAP plot of subclusters between BM-lung and GM. UMAP plot of macrophage subclusters between BM-lung and GM, color-coded based on their original datasets (A), UMAP plot of macrophage subclusters between BM-lung and GM, color-coded by their clusters (B), UMAP plot of CD4+ T cell between BM-lung and GM, color-coded based on their original datasets (C), UMAP plot of CD4+ T cell subclusters between BM-lung and GM, color-coded by their clusters (D), UMAP plot of CD8+ T cell between BM-lung and GM, color-coded based on their original datasets (E), UMAP plot of CD8+ T cell subclusters between BM-lung and GM, color-coded by their clusters (F).
(TIF)

S3 Fig UMAP plot of subclusters between BM- breast and GM. UMAP plot of macrophage subclusters between BM-breast and GM, color-coded based on their original datasets (A), UMAP plot of macrophage subclusters between BM-breast and GM, color-coded by their clusters (B), UMAP plot of cancer-associated fibroblast subclusters BM-breast and GM, color-coded based on their original datasets (C), UMAP plot of cancer-associated fibroblast subclusters between BM-breast and GM, color-coded by their clusters (D).
(TIF)

S1 File S1 Table. Percentage of each cell type in BM-lung and GM. S2 Table. Percentage of subclusters of macrophages in BM-lung and GM. S3 Table. Percentage of subclusters of CD4+ T cell in BM-lung and GM. S4 Table. Percentage of subclusters of CD8+ T cell in BM-lung and GM. S5 Table. Percentage of each cell type in BM-breast and GM. S6 Table. Percentage of subclusters of macrophages in BM-breast and GM. S7 Table. Percentage of subclusters of cancer associated fibroblast in BM-breast and GM.
(DOCX)

S2 File (RAR)

S1 Data The top 100 up-regulated genes for each cluster in BM-lung and GM analysis. (XLSX)

S2 Data The top 100 up-regulated genes for each cluster in BM- breast and GM analysis. (XLSX)

## References

[1] Cui X, Wang Q, Zhou J, Wang Y, Xu C, Tong F, et al. Single-Cell Transcriptomics of Glioblastoma Reveals a Unique Tumor Microenvironment and Potential Immunotherapeutic Target Against Tumor-Associated Macrophage. Front Oncol. 2021;11. doi: 10.3389/fonc.2021.710695 34434898 PMC8382282

[2] Seoane J, De Mattos-Arruda L. Brain metastasis: New opportunities to tackle therapeutic resistance. Molecular Oncology. John Wiley and Sons Ltd; 2014. pp. 1120–1131. doi: 10.1016/j.molonc.2014.05.009 24953014 PMC5528619

[3] Sun H, Li L, Lao I, Li X, Xu B, Cao Y, et al. Single‐cell RNA sequencing reveals cellular and molecular reprograming landscape of gliomas and lung cancer brain metastases. Clin Transl Med. 2022;12. doi: 10.1002/ctm2.1101 36336787 PMC9637666

[4] Karimi E, Yu MW, Maritan SM, Perus LJM, Rezanejad M, Sorin M, et al. Single-cell spatial immune landscapes of primary and metastatic brain tumours. Nature. 2023;614: 555–563. doi: 10.1038/s41586-022-05680-3 36725935 PMC9931580

[5] Wasilewski D, Priego N, Fustero-Torre C, Valiente M. Reactive astrocytes in brain metastasis. Frontiers in Oncology. Frontiers Media S.A.; 2017. doi: 10.3389/fonc.2017.00298 29312881 PMC5732246

[6] Wu Y, Kang K, Han C, Wang L, Wang Z, Zhao A. Single-Cell Profiling Comparisons of Tumor Microenvironment between Primary Advanced Lung Adenocarcinomas and Brain Metastases and Machine Learning Algorithms in Predicting Immunotherapeutic Responses. Biomolecules. 2023;13. doi: 10.3390/biom13010185 36671569 PMC9855438

[7] Zhang C, Yu D. Advances in decoding breast cancer brain metastasis. Cancer Metastasis Rev. 2016;35: 677–684. doi: 10.1007/s10555-016-9638-9 27873078 PMC5215972

[8] Gonzalez H, Mei W, Robles I, Hagerling C, Allen BM, Hauge Okholm TL, et al. Cellular architecture of human brain metastases. Cell. 2022;185: 729–745.e20. doi: 10.1016/j.cell.2021.12.043 35063085 PMC8857062

[9] Oja AE, Piet B, Van Der Zwan D, Blaauwgeers H, Mensink M, De Kivit S, et al. Functional heterogeneity of CD4+ tumor-infiltrating lymphocytes with a resident memory phenotype in NSCLC. Front Immunol. 2018;9: 1–15. doi: 10.3389/fimmu.2018.02654 30505306 PMC6250821

[10] Richardson JR, Schöllhorn A, Gouttefangeas C, Schuhmacher J. CD4+ T cells: Multitasking cells in the duty of cancer immunotherapy. Cancers (Basel). 2021;13: 1–19. doi: 10.3390/cancers13040596 33546283 PMC7913359

[11] Fordham AJ, Hacherl CC, Patel N, Jones K, Myers B, Abraham M, et al. Differentiating glioblastomas from solitary brain metastases: An update on the current literature of advanced imaging modalities. Cancers. MDPI; 2021. doi: 10.3390/cancers13122960 34199151 PMC8231515

[12] Xiao Y, Wang Z, Zhao M, Deng Y, Yang M, Su G, et al. Single-Cell Transcriptomics Revealed Subtype-Specific Tumor Immune Microenvironments in Human Glioblastomas. Front Immunol. 2022;13. doi: 10.3389/fimmu.2022.914236 35669791 PMC9163377

[13] Liu Yafei, Ye Guanchao, Huang Lan, Zhang Chunyang, Sheng Yinliang, Wu Bin, et al. Single-cell transcriptome analysis demonstrates inter-patient and intra-tumor heterogeneity in primary and metastatic lung adenocarcinoma. Aging (Albany NY). 2020;12: 21559–21581. doi: 10.18632/aging.103945 33170151 PMC7695431

[14] Biermann J, Melms JC, Amin AD, Wang Y, Caprio LA, Karz A, et al. Dissecting the treatment-naive ecosystem of human melanoma brain metastasis. Cell. 2022;185: 2591–2608.e30. doi: 10.1016/j.cell.2022.06.007 35803246 PMC9677434

[15] Jackson HW, Fischer JR, Zanotelli VRT, Ali HR, Mechera R, Soysal SD, et al. The single-cell pathology landscape of breast cancer. Nature. 2020;578: 615–620. doi: 10.1038/s41586-019-1876-x 31959985

[16] Di Giacomo AM, Valente M, Cerase A, Lofiego MF, Piazzini F, Calabrò L, et al. Immunotherapy of brain metastases: Breaking a “dogma.” Journal of Experimental and Clinical Cancer Research. BioMed Central Ltd.; 2019. doi: 10.1186/s13046-019-1426-2 31623643 PMC6798349

[17] Hernández Martínez A, Madurga R, García-Romero N, Ayuso-Sacido Á. Unravelling glioblastoma heterogeneity by means of single-cell RNA sequencing. Cancer Letters. Elsevier Ireland Ltd; 2022. pp. 66–79. doi: 10.1016/j.canlet.2021.12.008 34902524

[18] Ochocka N, Segit P, Walentynowicz KA, Wojnicki K, Cyranowski S, Swatler J, et al. Single-cell RNA sequencing reveals functional heterogeneity of glioma-associated brain macrophages. Nat Commun. 2021;12. doi: 10.1038/s41467-021-21407-w 33608526 PMC7895824

[19] Yu K, Hu Y, Wu F, Guo Q, Qian Z, Hu W, et al. Surveying brain tumor heterogeneity by single-cell RNA-sequencing of multi-sector biopsies. Natl Sci Rev. 2020;7: 1306–1318. doi: 10.1093/nsr/nwaa099 34692159 PMC8289159

[20] Abdelfattah N, Kumar P, Wang C, Leu JS, Flynn WF, Gao R, et al. Single-cell analysis of human glioma and immune cells identifies S100A4 as an immunotherapy target. Nat Commun. 2022;13. doi: 10.1038/s41467-022-28372-y 35140215 PMC8828877

[21] Wang L, Dai J, Han RR, Dong L, Feng D, Zhu G, et al. Single-Cell Map of Diverse Immune Phenotypes in the Metastatic Brain Tumor Microenvironment of Non Small Cell Lung Cancer. bioRxiv. 2019; 2019.12.30.890517. doi: 10.1101/2019.12.30.890517

[22] Quail DF, Joyce JA. The Microenvironmental Landscape of Brain Tumors. Cancer Cell. Cell Press; 2017. pp. 326–341. doi: 10.1016/j.ccell.2017.02.009 28292436 PMC5424263

[23] Osswald M, Jung E, Sahm F, Solecki G, Venkataramani V, Blaes J, et al. Brain tumour cells interconnect to a functional and resistant network. Nature. 2015;528: 93–98. doi: 10.1038/nature16071 26536111

[24] Sainz de Aja J, Dost AFM, Kim CF. Alveolar progenitor cells and the origin of lung cancer. J Intern Med. 2021;289: 629–635. doi: 10.1111/joim.13201 33340175 PMC8604037

[25] Wu SZ, Al-Eryani G, Roden DL, Junankar S, Harvey K, Andersson A, et al. A single-cell and spatially resolved atlas of human breast cancers. Nat Genet. 2021;53: 1334–1347. doi: 10.1038/s41588-021-00911-1 34493872 PMC9044823

[26] Yan X, Xie Y, Yang F, Hua Y, Zeng T, Sun C, et al. Comprehensive description of the current breast cancer microenvironment advancements via single-cell analysis. Journal of Experimental and Clinical Cancer Research. BioMed Central Ltd; 2021. doi: 10.1186/s13046-021-01949-z 33906694 PMC8077685

[27] Louis DN, Perry A, Wesseling P, Brat DJ, Cree IA, Figarella-Branger D, et al. The 2021 WHO classification of tumors of the central nervous system: A summary. Neuro Oncol. 2021;23: 1231–1251. doi: 10.1093/neuonc/noab106 34185076 PMC8328013

[28] Salehi N, Karimi-Jafari MH, Totonchi M, Amiri-Yekta A. Integration and gene co-expression network analysis of scRNA-seq transcriptomes reveal heterogeneity and key functional genes in human spermatogenesis. Sci Rep. 2021;11: 1–13. doi: 10.1038/s41598-021-98267-3 34580317 PMC8476490

[29] Li Q, Wang R, Yang Z, Li W, Yang J, Wang Z, et al. Molecular profiling of human non-small cell lung cancer by single-cell RNA-seq. Genome Med. 2022;14. doi: 10.1186/s13073-022-01089-9 35962452 PMC9375433

[30] Zhang J, Song C, Tian Y, Yang X. Single-Cell RNA Sequencing in Lung Cancer: Revealing Phenotype Shaping of Stromal Cells in the Microenvironment. Frontiers in Immunology. Frontiers Media S.A.; 2022. doi: 10.3389/fimmu.2021.802080 35126365 PMC8807562

[31] Kim N, Kim HK, Lee K, Hong Y, Cho JH, Choi JW, et al. Single-cell RNA sequencing demonstrates the molecular and cellular reprogramming of metastatic lung adenocarcinoma. Nat Commun. 2020;11. doi: 10.1038/s41467-020-16164-1 32385277 PMC7210975

[32] Tian Y, Li Q, Yang Z, Zhang S, Xu J, Wang Z, et al. Single-cell transcriptomic profiling reveals the tumor heterogeneity of small-cell lung cancer. Signal Transduct Target Ther. 2022;7. doi: 10.1038/s41392-022-01150-4 36195615 PMC9532437

[33] Wu F, Fan J, He Y, Xiong A, Yu J, Li Y, et al. Single-cell profiling of tumor heterogeneity and the microenvironment in advanced non-small cell lung cancer. Nat Commun. 2021;12. doi: 10.1038/s41467-021-22801-0 33953163 PMC8100173

[34] Travaglini KJ, Nabhan AN, Penland L, Sinha R, Gillich A, Sit R V., et al. A molecular cell atlas of the human lung from single-cell RNA sequencing. Nature. 2020;587: 619–625. doi: 10.1038/s41586-020-2922-4 33208946 PMC7704697

[35] Franks TJ, Colby T V., Travis WD, Tuder RM, Reynolds HY, Brody AR, et al. Resident Cellular Components of the Human Lung Current Knowledge and Goals for Research on Cell Phenotyping and Function. Proceedings of the American Thoracic Society. 2008. pp. 763–766. doi: 10.1513/pats.200803-025HR 18757314

[36] Fan XX, Wu Q. Decoding Lung Cancer at Single-Cell Level. Frontiers in Immunology. Frontiers Media S.A.; 2022. doi: 10.3389/fimmu.2022.883758 35677034 PMC9167930

[37] Tan Z, Kan C, Sun M, Yang F, Wong M, Wang S, et al. Mapping Breast Cancer Microenvironment Through Single-Cell Omics. Frontiers in Immunology. Frontiers Media S.A.; 2022. doi: 10.3389/fimmu.2022.868813 35514975 PMC9065352

[38] Xu L, Saunders K, Knutsdottir H, Chen K, Maués J, Hodgdon C, et al. A comprehensive single-cell breast tumor atlas defines cancer epithelial and immune cell heterogeneity and interactions predicting anti-PD-1 therapy response. bioRxiv. 2022; 2022.08.01.501918. doi: 10.1101/2022.08.01.501918 PMC11148512 38614094

[39] Ren L, Li J, Wang C, Lou Z, Gao S, Zhao L, et al. Single cell RNA sequencing for breast cancer: present and future. Cell Death Discovery. Springer Nature; 2021. doi: 10.1038/s41420-021-00485-1 33990550 PMC8121804

[40] Gambardella G, Viscido G, Tumaini B, Isacchi A, Bosotti R, di Bernardo D. A single-cell analysis of breast cancer cell lines to study tumour heterogeneity and drug response. Nat Commun. 2022;13. doi: 10.1038/s41467-022-29358-6 35361816 PMC8971486

[41] Chen YC, Sahoo S, Brien R, Jung S, Humphries B, Lee W, et al. Single-cell RNA-sequencing of migratory breast cancer cells: Discovering genes associated with cancer metastasis. Analyst. 2019;144: 7296–7309. doi: 10.1039/c9an01358j 31710321 PMC8942075

[42] Zou Y, Ye F, Kong Y, Hu X, Deng X, Xie J, et al. The Single-Cell Landscape of Intratumoral Heterogeneity and The Immunosuppressive Microenvironment in Liver and Brain Metastases of Breast Cancer. Adv Sci. 2023;10. doi: 10.1002/advs.202203699 36529697 PMC9929130

[43] Galbo PM, Liu Y, Peng M, Wei Y, Madsen AT, Graff S, et al. Functional Contribution of Cancer-Associated Fibroblasts in Glioblastoma. bioRxiv. 2022; 2022.04.07.487495. doi: 10.1101/2022.04.07.487495 PMC10922678 38060213

[44] Sebastian A, Hum NR, Martin KA, Gilmore SF, Peran I, Byers SW, et al. Single-cell transcriptomic analysis of tumor- derived fibroblasts and normal tissue-resident fibroblasts reveals fibroblast heterogeneity in breast cancer. Cancers (Basel). 2020;12. doi: 10.3390/cancers12051307 32455670 PMC7281266

[45] Cords L, Tietscher S, Anzeneder T, Langwieder C, Rees M, de Souza N, et al. A Cancer-Associated Fibroblast Classification Framework for Single-Cell Data. bioRxiv. 2022; 2022.12.14.520398. doi: 10.1101/2022.12.14.520398 PMC10354071 37463917

[46] Hu L, Su L, Cheng H, Mo C, Ouyang T, Li J, et al. Single-cell RNA sequencing reveals the cellular origin and evolution of breast cancer in BRCA1 mutation carriers. Cancer Res. 2021;81: 2600–2611. doi: 10.1158/0008-5472.CAN-20-2123 33727227

[47] Brown CE, Alizadeh D, Starr R, Weng L, Wagner JR, Naranjo A, et al. Regression of Glioblastoma after Chimeric Antigen Receptor T-Cell Therapy. N Engl J Med. 2016;375: 2561–2569. doi: 10.1056/NEJMoa1610497 28029927 PMC5390684

[48] Wu M, Liang Y, Zhang X. Changes in Pulmonary Microenvironment Aids Lung Metastasis of Breast Cancer. Frontiers in Oncology. Frontiers Media S.A.; 2022. doi: 10.3389/fonc.2022.860932 35719975 PMC9204317

[49] Singh R, Mishra MK, Aggarwal H. Inflammation, Immunity, and Cancer. Mediators of Inflammation. Hindawi Limited; 2017. doi: 10.1155/2017/6027305 29234189 PMC5695028

[50] Grivennikov SI, Greten FR, Karin M. Immunity, Inflammation, and Cancer. Cell. 2010. pp. 883–899. doi: 10.1016/j.cell.2010.01.025 20303878 PMC2866629

[51] Dunn GP, Old LJ, Schreiber RD. The three Es of cancer immunoediting. Annual Review of Immunology. 2004. pp. 329–360. doi: 10.1146/annurev.immunol.22.012703.104803 15032581

[52] Thommen DS, Schumacher TN. T Cell Dysfunction in Cancer. Cancer Cell. Cell Press; 2018. pp. 547–562. doi: 10.1016/j.ccell.2018.03.012 29634943 PMC7116508

[53] Zhou S, Huang Y e., Liu H, Zhou X, Yuan M, Hou F, et al. Single-cell RNA-seq dissects the intratumoral heterogeneity of triple-negative breast cancer based on gene regulatory networks. Mol Ther—Nucleic Acids. 2021;23: 682–690. doi: 10.1016/j.omtn.2020.12.018 33575114 PMC7851423

[54] Pal B, Chen Y, Vaillant F, Capaldo BD, Joyce R, Song X, et al. A single‐cell RNA expression atlas of normal, preneoplastic and tumorigenic states in the human breast. EMBO J. 2021;40: 105–108. doi: 10.15252/embj.2020107333 33950524 PMC8167363

[55] Klemm F, Maas RR, Bowman RL, Kornete M, Soukup K, Nassiri S, et al. Interrogation of the Microenvironmental Landscape in Brain Tumors Reveals Disease-Specific Alterations of Immune Cells. Cell. 2020;181: 1643–1660.e17. doi: 10.1016/j.cell.2020.05.007 32470396 PMC8558904

[56] Rubio-Perez C, Planas-Rigol E, Trincado JL, Bonfill-Teixidor E, Arias A, Marchese D, et al. Immune cell profiling of the cerebrospinal fluid enables the characterization of the brain metastasis microenvironment. Nat Commun. 2021;12. doi: 10.1038/s41467-021-21789-x 33686071 PMC7940606

[57] Mirzaei R, Yong VW. Microglia–T cell conversations in brain cancer progression. Trends Mol Med. 2022;28: 951–963. doi: 10.1016/j.molmed.2022.08.006 36075812

[58] Friebel E, Kapolou K, Unger S, Núñez NG, Utz S, Rushing EJ, et al. Single-Cell Mapping of Human Brain Cancer Reveals Tumor-Specific Instruction of Tissue-Invading Leukocytes. Cell. 2020;181: 1626–1642.e20. doi: 10.1016/j.cell.2020.04.055 32470397

[59] Zhang Z, Liu S, Zhang B, Qiao L, Zhang Y, Zhang Y. T Cell Dysfunction and Exhaustion in Cancer. Frontiers in Cell and Developmental Biology. Frontiers Media S.A.; 2020. doi: 10.3389/fcell.2020.00017 32117960 PMC7027373

[60] Guo X, Zhang Y, Zheng L, Zheng C, Song J, Zhang Q, et al. Global characterization of T cells in non-small-cell lung cancer by single-cell sequencing. Nat Med. 2018;24: 978–985. doi: 10.1038/s41591-018-0045-3 29942094

[61] Sudmeier LJ, Hoang KB, Nduom EK, Wieland A, Neill SG, Schniederjan MJ, et al. Distinct phenotypic states and spatial distribution of CD8+ T cell clonotypes in human brain metastases. Cell Reports Med. 2022;3: 100620. doi: 10.1016/j.xcrm.2022.100620 35584630 PMC9133402

[62] Tietscher S, Wagner J, Anzeneder T, Langwieder C, Rees M, Sobottka B, et al. A comprehensive single-cell map of T cell exhaustion-associated immune environments in human breast cancer. Nat Commun. 2023;14. doi: 10.1038/s41467-022-35238-w 36609566 PMC9822999

[63] Deng W, Ma Y, Su Z, Liu Y, Liang P, Huang C, et al. Single-cell RNA-sequencing analyses identify heterogeneity of CD8+ T cell subpopulations and novel therapy targets in melanoma. Mol Ther—Oncolytics. 2021;20: 105–118. doi: 10.1016/j.omto.2020.12.003 33575475 PMC7851490

[64] Orimo A, Gupta PB, Sgroi DC, Arenzana-Seisdedos F, Delaunay T, Naeem R, et al. Stromal fibroblasts present in invasive human breast carcinomas promote tumor growth and angiogenesis through elevated SDF-1/CXCL12 secretion. Cell. 2005;121: 335–348. doi: 10.1016/j.cell.2005.02.034 15882617

[65] Zeltz C, Primac I, Erusappan P, Alam J, Noel A, Gullberg D. Cancer-associated fibroblasts in desmoplastic tumors: emerging role of integrins. Semin Cancer Biol. 2020;62: 166–181. doi: 10.1016/j.semcancer.2019.08.004 31415910

[66] Li H, Courtois ET, Sengupta D, Tan Y, Chen KH, Goh JJL, et al. Reference component analysis of single-cell transcriptomes elucidates cellular heterogeneity in human colorectal tumors. Nat Genet. 2017;49: 708–718. doi: 10.1038/ng.3818 28319088

[67] Friedman G, Levi-Galibov O, David E, Bornstein C, Giladi A, Dadiani M, et al. Cancer-associated fibroblast compositions change with breast cancer progression linking the ratio of S100A4+ and PDPN+ CAFs to clinical outcome. Nat Cancer. 2020;1: 692–708. doi: 10.1038/s43018-020-0082-y 35122040 PMC7617059

[68] Hu H, Piotrowska Z, Hare PJ, Chen H, Mulvey HE, Mayfield A, et al. Three subtypes of lung cancer fibroblasts define distinct therapeutic paradigms. Cancer Cell. 2021;39: 1531–1547.e10. doi: 10.1016/j.ccell.2021.09.003 34624218 PMC8578451

[69] Pelon F, Bourachot B, Kieffer Y, Magagna I, Mermet-Meillon F, Bonnet I, et al. Cancer-associated fibroblast heterogeneity in axillary lymph nodes drives metastases in breast cancer through complementary mechanisms. Nat Commun. 2020;11. doi: 10.1038/s41467-019-14134-w 31964880 PMC6972713

[70] Costa A, Kieffer Y, Scholer-Dahirel A, Pelon F, Bourachot B, Cardon M, et al. Fibroblast Heterogeneity and Immunosuppressive Environment in Human Breast Cancer. Cancer Cell. 2018;33: 463–479.e10. doi: 10.1016/j.ccell.2018.01.011 29455927

[71] Bartoschek M, Oskolkov N, Bocci M, Lövrot J, Larsson C, Sommarin M, et al. Spatially and functionally distinct subclasses of breast cancer-associated fibroblasts revealed by single cell RNA sequencing. Nat Commun. 2018;9. doi: 10.1038/s41467-018-07582-3 30514914 PMC6279758

[72] Tay RE, Richardson EK, Toh HC. Revisiting the role of CD4+ T cells in cancer immunotherapy—new insights into old paradigms. Cancer Gene Ther. 2021;28: 5–17. doi: 10.1038/s41417-020-0183-x 32457487 PMC7886651

[73] Clavreul A, Guette C, Faguer R, Tétaud C, Boissard A, Lemaire L, et al. Glioblastoma-associated stromal cells (GASCs) from histologically normal surgical margins have a myofibroblast phenotype and angiogenic properties. J Pathol. 2014;233: 74–88. doi: 10.1002/path.4332 24481573

[74] Mhaidly R, Mechta-Grigoriou F. Role of cancer-associated fibroblast subpopulations in immune infiltration, as a new means of treatment in cancer. Immunol Rev. 2021;302: 259–272. doi: 10.1111/imr.12978 34013544 PMC8360036

[75] Lim ZF, Ma PC. Emerging insights of tumor heterogeneity and drug resistance mechanisms in lung cancer targeted therapy. J Hematol Oncol. 2019;12: 1–18. doi: 10.1186/s13045-019-0818-2 31815659 PMC6902404

[76] Lovly CM, Salama AKS, Salgia R. Tumor Heterogeneity and Therapeutic Resistance. Am Soc Clin Oncol Educ B. 2016;36: e585–e593. doi: 10.1200/EDBK_158808 27249771 PMC10132823

[77] Marino FZ, Bianco R, Accardo M, Ronchi A, Cozzolino I, Morgillo F, et al. Molecular heterogeneity in lung cancer: From mechanisms of origin to clinical implications. Int J Med Sci. 2019;16: 981–989. doi: 10.7150/ijms.34739 31341411 PMC6643125

[78] Zhu L, Jiang M, Wang H, Sun H, Zhu J, Zhao W, et al. A narrative review of tumor heterogeneity and challenges to tumor drug therapy. Ann Transl Med. 2021;9: 1351–1351. doi: 10.21037/atm-21-1948 34532488 PMC8422119

[79] Jiménez-Sánchez A, Cybulska P, Mager KLV, Koplev S, Cast O, Couturier DL, et al. Unraveling tumor–immune heterogeneity in advanced ovarian cancer uncovers immunogenic effect of chemotherapy. Nat Genet. 2020;52: 582–593. doi: 10.1038/s41588-020-0630-5 32483290 PMC8353209

[80] Heys SD, Stewart KN, McKenzie EJ, Miller ID, Wong SYC, Sellar G, et al. Characterisation of tumour-infiltrating macrophages: Impact on response and survival in patients receiving primary chemotherapy for breast cancer. Breast Cancer Res Treat. 2012;135: 539–548. doi: 10.1007/s10549-012-2190-6 22886449

[81] Massa C, Karn T, Denkert C, Schneeweiss A, Hanusch C, Blohmer JU, et al. Differential effect on different immune subsets of neoadjuvant chemotherapy in patients with TNBC. J Immunother Cancer. 2020;8. doi: 10.1136/jitc-2020-001261 33199511 PMC7670944

[82] Larionova I, Cherdyntseva N, Liu T, Patysheva M, Rakina M, Kzhyshkowska J. Interaction of tumor-associated macrophages and cancer chemotherapy. Oncoimmunology. 2019;8: 1–15. doi: 10.1080/2162402X.2019.1596004 31143517 PMC6527283

[83] Lv X, Mao Z, Sun X, Liu B. Intratumoral Heterogeneity in Lung Cancer. Cancers (Basel). 2023;15. doi: 10.3390/cancers15102709 37345046 PMC10216154

[84] Junttila MR, De Sauvage FJ. Influence of tumour micro-environment heterogeneity on therapeutic response. Nature. 2013;501: 346–354. doi: 10.1038/nature12626 24048067

