# Supplementary figures and images for: Comprehensive integrated single-cell RNA sequencing analysis of brain metastasis and glioma microenvironment: Contrasting heterogeneity landscapes
Source: PLoS One. 2024 Jul 26;19(7):e0306220. doi: 10.1371/journal.pone.0306220 (PMC11280140; doi:10.1371/journal.pone.0306220)

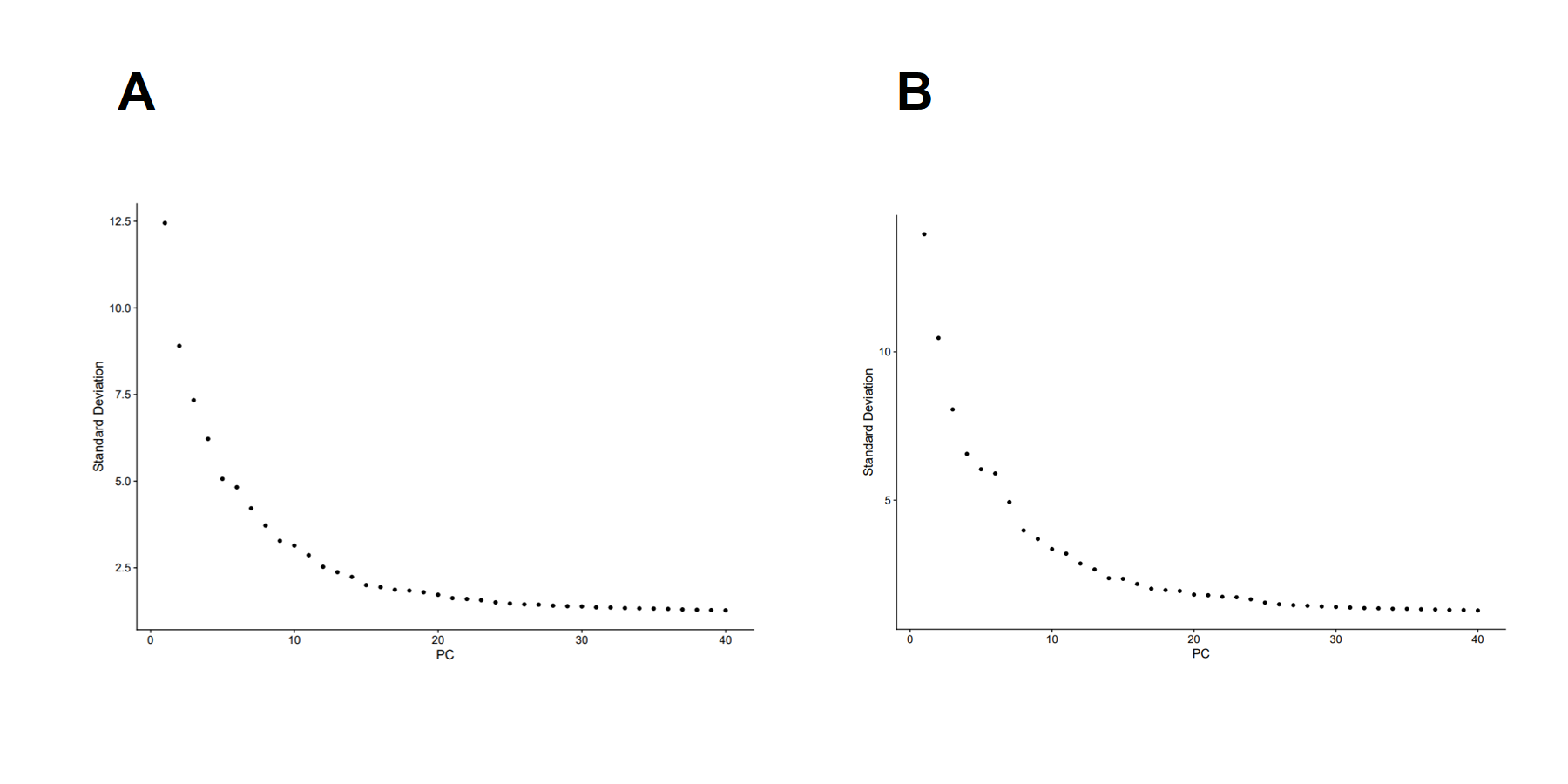

Supplement: S1 Fig — Elbow plot of BM-lung and GM (A) Elbow plot of BM- breast and GM (B). (TIF) [file pone.0306220.s001.tif]

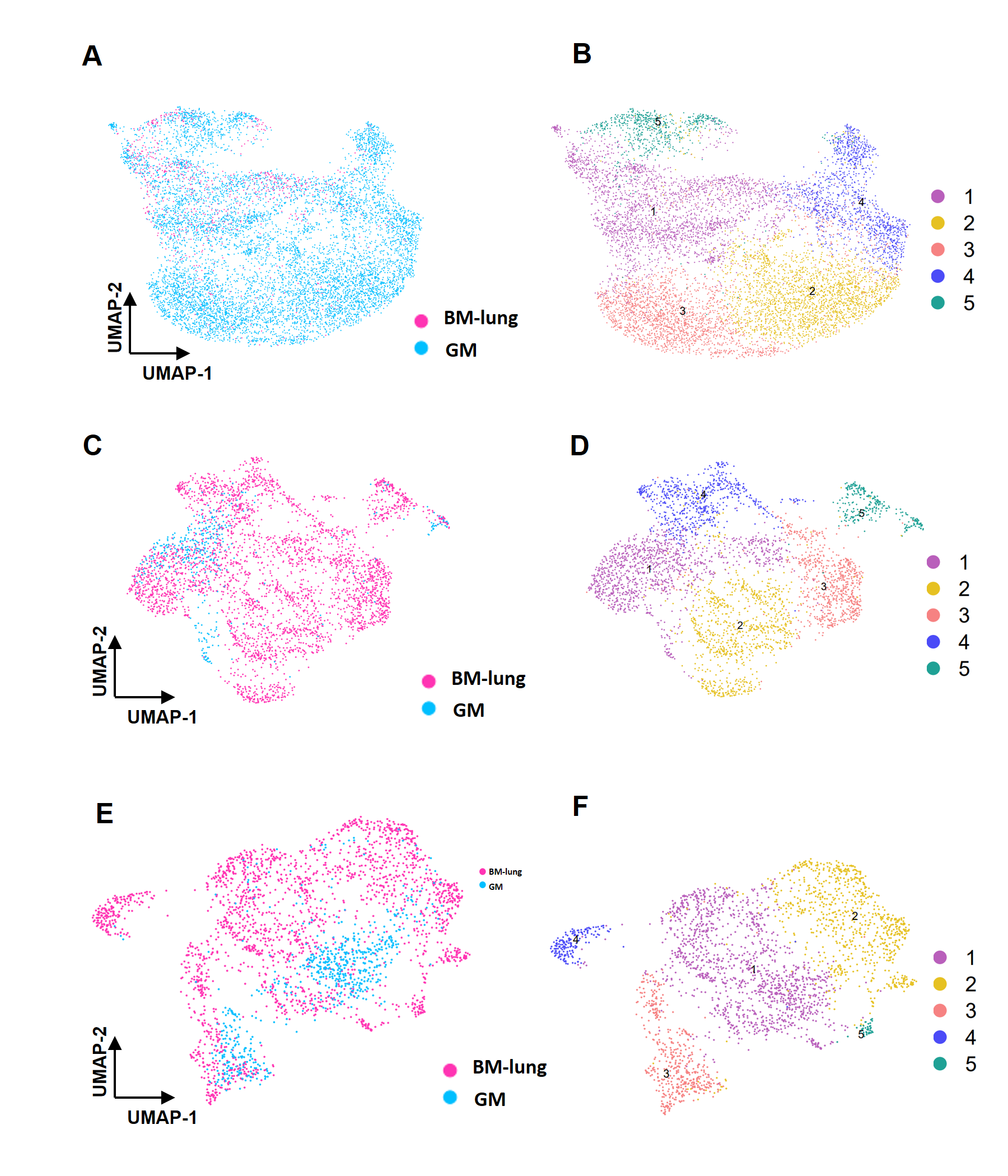

Supplement: S2 Fig — UMAP plot of macrophage subclusters between BM-lung and GM, color-coded based on their original datasets (A), UMAP plot of macrophage subclusters between BM-lung and GM, color-coded by their clusters (B), UMAP plot of CD4+ T cell between BM-lung and GM, color-coded based on their original datasets (C), UMAP plot of CD4+ T cell subclusters between BM-lung and GM, color-coded by their clusters (D), UMAP plot of CD8+ T cell between BM-lung and GM, color-coded based on their original datasets (E), UMAP plot of CD8+ T cell subclusters between BM-lung and GM, color-coded by their clusters (F). (TIF) [file pone.0306220.s002.tif]

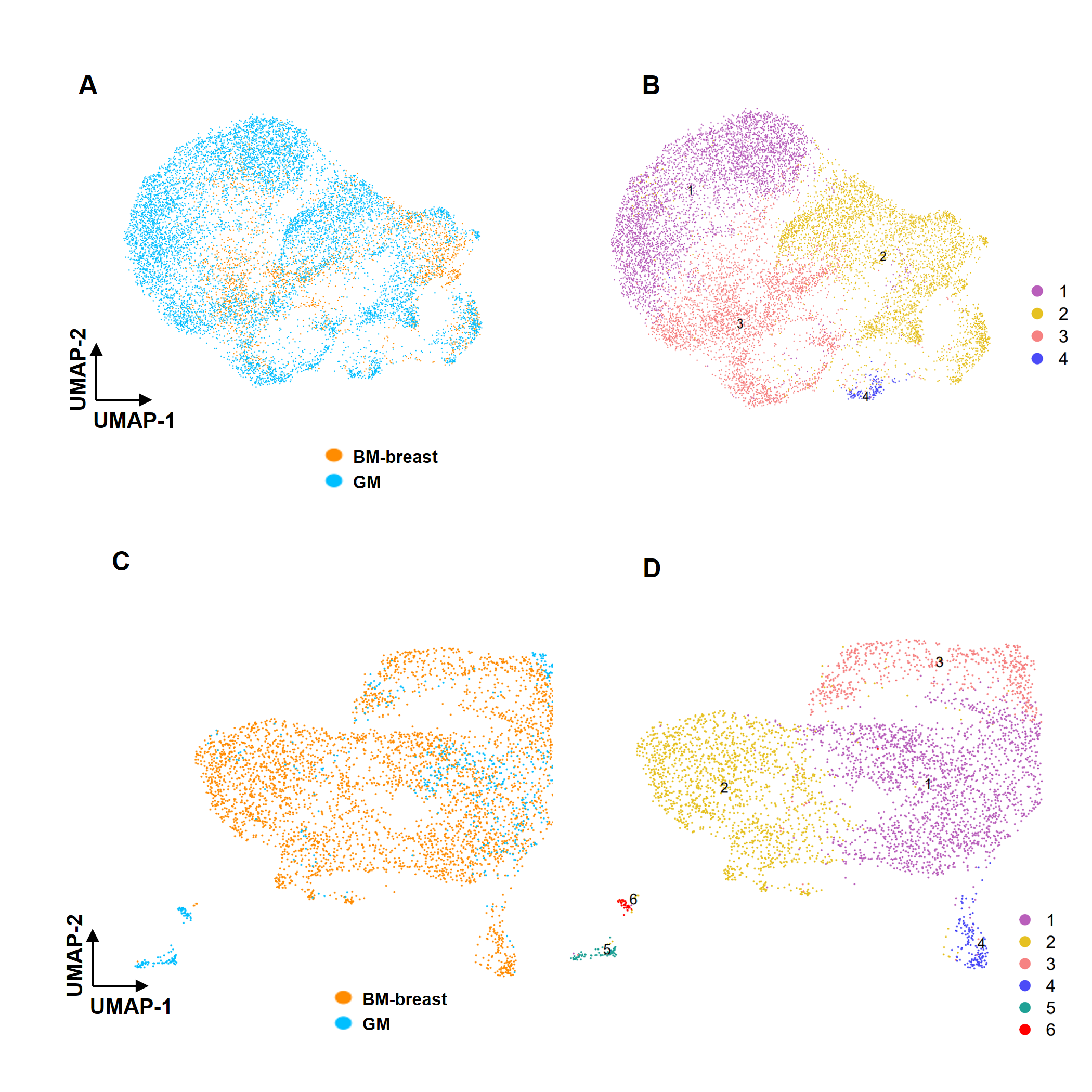

Supplement: S3 Fig — UMAP plot of macrophage subclusters between BM-breast and GM, color-coded based on their original datasets (A), UMAP plot of macrophage subclusters between BM-breast and GM, color-coded by their clusters (B), UMAP plot of cancer-associated fibroblast subclusters BM-breast and GM, color-coded based on their original datasets (C), UMAP plot of cancer-associated fibroblast subclusters between BM-breast and GM, color-coded by their clusters (D). (TIF) [file pone.0306220.s003.tif]
