# Supplementary material for: Comprehensive integrated single-cell RNA sequencing analysis of brain metastasis and glioma microenvironment: Contrasting heterogeneity landscapes
Source: PLoS One. 2024 Jul 26;19(7):e0306220. doi: 10.1371/journal.pone.0306220 (PMC11280140; doi:10.1371/journal.pone.0306220)
Supplement: S1 File — Percentage of each cell type in BM-lung and GM. S2 Table. Percentage of subclusters of macrophages in BM-lung and GM. S3 Table. Percentage of subclusters of CD4+ T cell in BM-lung and GM. S4 Table. Percentage of subclusters of CD8+ T cell in BM-lung and GM. S5 Table. Percentage of each cell type in BM-breast and GM. S6 Table. Percentage of subclusters of macrophages in BM-breast and GM. S7 Table. Percentage of subclusters of cancer associated fibroblast in BM-breast and GM. (DOCX) [file pone.0306220.s004.docx]

**S1 Table**: percentage of each cell type in BM-lung and GM.

| cell types | clusters | GM | BM-lung |
| --- | --- | --- | --- |
| epithelial | 1 | 13.55422777 | 27.93772659 |
| macrophage | 2 | 30.04888647 | 3.039027511 |
| proliferating | 3 | 6.007604563 | 15.77450771 |
| epithelial (AT2) | 4 | 2.053231939 | 13.02694249 |
| oligodendrocyte | 5 | 13.59768242 | 1.439539347 |
| CD4+ T cell | 6 | 1.524533768 | 11.86464776 |
| astrocyte | 7 | 9.288430201 | 2.381460155 |
| CD8+ T cell | 8 | 2.505884483 | 7.890808275 |
| B cell | 9 | 5.268875611 | 4.318618042 |
| fibroblast | 10 | 1.95545899 | 5.392052321 |
| OPC | 11 | 6.268332428 | 0.170612071 |
| monocyte | 12 | 4.722071338 | 0.017772091 |
| undetermined | 13 | 0.706137968 | 3.881424611 |
| endothelial | 14 | 1.281912004 | 1.709675126 |
| dendritic cell | 15 | 1.216730038 | 1.155185896 |

**S2 Table**: percentage of subclusters of macrophages in BM-lung and GM

| cell types | clusters | GM | BM-lung |
| --- | --- | --- | --- |
| MDM1 | 1 | 25.43987 | 71.22807 |
| MG1 | 2 | 27.76573 | 4.327485 |
| MG2 | 3 | 24.53603 | 8.421053 |
| MG3 | 4 | 15.01567 | 4.678363 |
| MDM2 | 5 | 7.242709 | 11.34503 |

**S3 Table**: percentage of subclusters of CD4+ T cell in BM-lung and GM

| cell types | clusters | GM | BM-lung |
| --- | --- | --- | --- |
| naive | 1 | 63.42043 | 27.77112 |
| Cytotoxic1 | 2 | 6.175772 | 26.60276 |
| Cytotoxic2 | 3 | 1.662708 | 21.65968 |
| T regulatory (Treg) | 4 | 19.47743 | 16.89635 |
| proliferating | 5 | 9.263658 | 7.070102 |

**S4 Table**: percentage of subclusters of CD8+ T cell in BM-lung and GM

| cell types | clusters | GM | BM-lung |
| --- | --- | --- | --- |
| Naive1 | 1 | 58.81503 | 43.33333 |
| Naive2 | 2 | 17.19653 | 33.1982 |
| cytotoxic | 3 | 23.55491 | 13.96396 |
| exhausted | 4 | 0.433526 | 7.657658 |
| proliferating | 5 | 0 | 1.846847 |

**S5 Table**: percentage of each cell type in BM-breast and GM

| \| Cell types \| clusters \| GM \| BM-breast \| \| --- \| --- \| --- \| --- \| \| epithelial \| 1 \| 5.308709 \| 49.19375926 \| \| macrophage \| 2 \| 32.42079 \| 6.432493681 \| \| OPC \| 3 \| 12.73945 \| 9.669078125 \| \| proliferating \| 4 \| 6.300923 \| 10.87480752 \| \| fibroblast \| 5 \| 2.169111 \| 11.26412737 \| \| oligodendrocyte \| 6 \| 13.20297 \| 0.679857056 \| \| B cell \| 7 \| 9.1979 \| 1.531131061 \| \| T cell \| 8 \| 3.889191 \| 3.009965426 \| \| astrocyte \| 9 \| 3.997827 \| 2.33010837 \| \| endothelial \| 10 \| 1.995292 \| 3.747929922 \| \| undetermined \| 11 \| 4.026797 \| 1.101136 \| \| monocyte \| 12 \| 4.751041 \| 0.165606206 \| |  |  |  |
| --- | --- | --- | --- | --- | --- | --- | --- | --- | --- | --- | --- | --- | --- | --- | --- | --- | --- | --- | --- | --- | --- | --- | --- | --- | --- | --- | --- | --- | --- | --- | --- | --- | --- | --- | --- | --- | --- | --- | --- | --- | --- | --- | --- | --- | --- | --- | --- | --- | --- | --- | --- | --- | --- | --- | --- |
| **S6 Table**: percentage of subclusters of macrophages in BM-breast and GM |  |  |  |
| \| Cell types \| clusters \| GM \| BM-breast \| \| --- \| --- \| --- \| --- \| \| MG \| 1 \| 45.2474 \| 10.56911 \| \| MDM1 \| 2 \| 30.51491 \| 40.92141 \| \| MDM2 \| 3 \| 22.6963 \| 48.14815 \| \| proliferating \| 4 \| 1.541383 \| 0.361337 \| |  |  |  |

|  |  |  |  |
| --- | --- | --- | --- |
| **S7 Table**: percentage of subclusters of cancer associated fibroblast in BM-breast and GM   \| Cell type \| Clusters \| GM \| BM-breast \| \| --- \| --- \| --- \| --- \| \| myCAF1 \| 1 \| 54.59098497 \| 42.300748 \| \| myCAF2 \| 2 \| 9.515859766 \| 40.72736652 \| \| iCAF \| 3 \| 16.86143573 \| 13.51560485 \| \| dCAF \| 4 \| 1.335559265 \| 3.456280629 \| \| apCAF \| 5 \| 11.18530885 \| 0 \| \| TpCAF \| 6 \| 6.510851419 \| 0 \| |  |  |  |
|  |  |  |  |
|  |  |  |  |
|  |  |  |  |
|  |  |  |  |
|  |  |  |  |
|  |  |  |  |
|  |  |  |  |
|  |  |  |  |
